# Editorial: Elimination of biliary atresia

**DOI:** 10.3389/fped.2023.1202727

**Published:** 2023-05-22

**Authors:** Magd Ahmed Kotb, Satoshi Ieiri, Sherif Mohamed Shehata

**Affiliations:** ^1^ Department of Pediatrics, Faculty of Medicine, Cairo University, Cairo, Egypt; ^2^ Department of Pediatric Surgery, Research Field in Medical and Health Sciences, Medical and Dental Area, Research and Education Assembly, Kagoshima University, Kagoshima, Japan; ^3^ Department of Pediatric Surgery, Faculty of Medicine, Tanta University, Tanta, Egypt

**Keywords:** biliary atresia, neonatal screening, Kotb disease, microchimerism, glutathione S transferase (GST), morphogenesis

**Editorial on the Research Topic** Elimination of biliary atresia

Biliary atresia (BA) is the most common indication for liver transplantation among children globally (1). This Research Topic aimed to shed light on the BA possible etiology(s), pathogenesis, underlying genetic susceptibility, screening, diagnosis, and challenges that need to be addressed for its elimination and eradication.

## The multifactorial etiology and genetic susceptibility to biliary atresia

BA proved to be a phenotype. Irrespective of the triggering factors, pathogenesis involves inflammation within the walls of the biliary system, a massive immune response, adhesions, and fibrous obliteration of biliary radicals. Accelerated cirrhosis is another constant feature of BA (2). Many factors have been flagged as the initial trigger of bile duct wall inflammation in the susceptible host: these include viral, vascular, immune, toxins as biliatresone (3), and aflatoxins. The aflatoxin-induced cholangiopathy in glutathione S transferase (GST) M1 deficient Egyptian neonates born to GST M1 heterozygous mothers is a known etiology of BA, namely, Kotb disease (4–6).

The known susceptibility factors include the congenital detoxification detect (4–6), the degree, severity and rate of the massive immune response, the genetic susceptibility to fibrosis and scaring, maternal microchimerism, co-existing viral infections as cytomegalovirus (7), or bacterial infection as *E coli* and others (5). The immune response involves neutrophil elastase (6, 8), CD4+, CD68+, CD8+2, CD14+, and others (2). Yang and coworkers have provided evidence that the immune response is dictated by genetic disposition. Immune dysregulation derived by maternal microchimerism seems to play another important role in the progress of the disease.

The multifactorial etiology of BA is not limited to genetic susceptibility to immune dysregulation or detoxification defects such as GST M1 deficiency or fibrinogenesis. There are no reports of BA in children with Down syndrome (9); hence, it seems that there are genetic protective sentinels against BA that remain to be explored. This protective gene(s) might be the focus of future research and may be usable within gene therapy. The incidence of BA is influenced by ethnicity and geographic location, providing more evidence to the multifactorial etiology. It provides insight that there is inherent protective and/or susceptibility genetic make-up triggered by environmental influencing factors.

The multifactorial etiology underscores that the strategies of screening and elimination might need to be individualized according to the local cause of BA in any given geographic area.

## Neonatal screening and early diagnosis of biliary atresia

The poor outcome of biliary atresia if management is delayed beyond the earliest 3 months of life makes BA a neonatal diagnostic emergency. The clay-colored stools in BA are easily noted by the parents and care givers. Hence, the clay stools are the basis for neonatal screening. Neonatal screening for BA by stool card (10–14) and by measurement of conjugated bilirubin has been associated with a reduction in late referrals, younger age at portoenterostomy, and a significant increase of the 5-year jaundice-free survival rate with own native liver. Yet, the final diagnosis relies upon invasive liver biopsy findings and intra-operative cholangiography. Less invasive indocyanine green cholangiography was reported to be successful in BA diagnosis, but its diagnostic sensitivity and specificity need to be studied. Thus far, the lack of a unanimous single gene defect among all ethnicities and geographical areas makes it difficult to implement a universal genetic testing tool for BA.

## Palliative management in biliary atresia and its disappointing outcome

There is no definitive curative treatment for BA. The standard management is palliative Kasai portoenterostomy to remove the obstruction and ensure bile flow into the intestine. Kasai portoenterostomy within the earliest 60–90 days of life is the standard treatment, and almost 75% will eventually need liver transplantation (1, 15). Predictors of a poor outcome have been serotyped as older age at operation, yet the work by Sun and co-workers provides evidence that the younger neonates with lower than expected gamma glutamyl transpeptidase are at higher risk for a poor outcome. Again, there is no single predictive factor for outcome in BA.

Immune involvement is fundamental to the development of the BA phenotype (4, 16), yet the immune modulatory therapies remain adjuvant to the surgical intervention (17). And despite the central role of immune pathogenesis, the roles of current immune modulatory therapies as steroids (18), immunoglobulins (19), colchicine (20), etc. are controversial. The inconsistency might be related to the late institution of therapy or lack of addressing the cause that triggered the massive immune response in BA. Choleresis by bile acids is used off-label in BA and is hepatotoxic and equally disappointing (21).

Despite the recent advances in our understanding of BA etiology and pathogenesis, effective curative treatment is not yet available. This might be attributed to the early onset of fibrosis and accelerated cirrhosis; as the cascade leading to fibrosis is almost always initiated prior to diagnosis. Currently, the march is not halted once the cascade of immune response and fibrosis sets in. The need for effective treatment seems to be related to timing at diagnosis. Genetic testing for GST M1 or other genes related to morphogenesis, angiogenesis and inflammatory pathways might be a goal for future neonatal screening for BA. It is not clear if the institution of immunomodulatory or chelation therapy early within the first 4 weeks of life of those with BA would make a difference in outcome before the development of fibrous adhesions of the extra-hepatic biliary system.

## Conclusion

Potential strategies to reduce the global burden of BA will be directed by future research. The future research areas needed to fill the knowledge gap include the following: the potential of genetic neonatal screening according to ethnicity and geographically known susceptibility genes, the outcome of BA if immune-modulatory therapy is instituted within the earliest 2–4 weeks, the role of enzyme replacement of missing enzymes as GSTM1, defining the protective gene against BA in patients with Down syndrome, defining if this protective gene is amenable to gene therapy among the other children with BA, the role of environmental control of aflatoxin contamination of foods, the role of abandoning milking of the umbilical cord at delivery, and the ideal timing and type of immune modulatory medicine for BA. More insight into BA susceptibility, etiology, screening, management, natural history, treatment, transplantation challenges, outcome, burden, and prevention is needed to plan BA eradication.
